# Trends in Psychiatric Emergency Department Visits in Northern Israel During the COVID-19 Outbreak

**DOI:** 10.3389/fpsyt.2021.603318

**Published:** 2021-07-20

**Authors:** Yael Pikkel Igal, Irit Meretyk, Aziz Darawshe, Samer Hayek, Limor Givon, Avi Levy, Idan Sipori, Yonatan Nuriel, Boaz Bloch, Shraga Buniak, Ron Eshel, Eyal Fruchter

**Affiliations:** ^1^Rambam Health Care Campus and Faculty of Medicine, Technion - Israel Institute of Technology, Haifa, Israel; ^2^Emek Medical Center, Technion - Israel Institute of Technology, Haifa, Israel; ^3^Meuhedet Health Services, Tel Aviv-Yafo, Israel; ^4^Tel-Aviv University, Tel Aviv-Yafo, Israel

**Keywords:** COVID-19, mental health, psychiatric emergency department visits, suicide, health seeking behavior

## Abstract

**Background:** During the spread of coronavirus disease (COVID-19), mandatory quarantines increased social isolation and anxiety, with inevitable consequences on mental health and health seeking behavior. We wished to estimate those trends.

**Methods:** We examined all psychiatric visits to the emergency department (ED) during March, April 2020, compared to identical months in 2018, 2019. We evaluated both number and nature of referrals.

**Results:** Throughout the years, psychiatric referrals comprised about 5% of the total number of ED visits. In March-April 2020, 30% decreases were observed in overall ED visits and in psychiatric referrals in the ED. Compared to 2018–2019, in 2020, the proportions of these diagnoses were higher: anxiety disorders (14.5 vs. 5.4%, *p* < 0.001), personality disorders (6.7 vs. 3.2%, *p* = 0.001), psychosis (9.5 vs. 6.7%, *p* = 0.049), post-traumatic stress disorder (3.2 vs. 1.5%, *p* = 0.023). Compared to 2018–2019, in 2020, proportions were lower for adjustment disorder (5.8 vs. 8.9%, *p* = 0.036) and for consultation regarding observation (11.7 vs. 31.6%, *p* < 0.001). Differences were not observed between 2018-2019 and 2020 in the proportions of other diagnoses including suicide and self-harm disorders. Referrals concerning suicide and self-harm in a rural hospital and community clinic were 30% lower in the COVID-19 lockdown than in the same months in 2018, 2019.

**Conclusion:** Psychiatric ED visits decreased by the same proportion as overall visits to the ED, apparently driven by fears of COVID-19. Referrals relating suicidality and self-harm shown nominal decrease, but their proportioned share remained constant. Increased anxiety and delayed care may eventually lead to increased mental health needs.

## Introduction

On December 2019, a chain of events led to the spread of coronavirus disease (COVID-19), which originated from Wuhan, People's Republic of China. COVID-19 is an infectious disease caused by a newly discovered coronavirus, which may be asymptomatic or cause mild to severe respiratory illness. Older populations and those with underlying medical conditions are more likely to develop serious illness, resulting in respiratory failure and possibly death (1). As of August 29 2020, there were 24,587,513 confirmed infections and 833,556 deaths globally^1^. In Israel, we are currently in the midst of a second wave of COVID-19 outbreak, with 112,000 confirmed patients and 894 deceased as of August 29, 2020^2^. In most countries in the world, a mandatory quarantine was enforced for varying periods, and hospitals transitioned to work in an emergency state.

Previous epidemics of coronavirus, such as the severe acute respiratory syndrome (SARS), had an interesting influence on health seeking behavior. Research conducted in the Taipei Veterans General Hospital showed that during the peak of the SARS epidemic in 2003, the reduction in daily emergency department (ED) visits reached 51.6% of pre-epidemic numbers (2). This finding was demonstrated in several researches; some found a decrease in trauma-related patients, with an increasing number of patients showing a chief complaint such as fever and respiratory symptoms (3). One hypothesis suggested to support this finding was that the fear of SARS reduced patient's health seeking behavior (4). Presumably, a pandemic, such as the current one, may change the way people respond to medical urgent care needs, both physical and psychiatric emergencies. The fear of COVID-19 may make people think twice before attending the ED and being evaluated. Consequences on mental health are potentially frightening and are yet to be determined, but several speculations have been made. One possible primary outcome is increased fear and anxiety, some of these may be distorted. This in turn might cause distress reactions such as insomnia, negative behaviors such as substance use, new onset of mental health disorders such as post-traumatic stress disorder (5–7), and the deterioration of the current condition of persons with psychiatric disorders. Medical and economic distress are also consequences of the pandemic crises, such medical issues could be COVID-19 sequalae such as neurological and pulmonary damage. Medical conditions that are not addressed as usual due to fears among those affected can eventually cause mental health problems, secondary to physical deterioration. Unemployment and peoples' fear of their economic future are conditions known to cause depression and anxiety, and even suicide (8).

Rambam Health Care Campus is the only referral hospital for northern Israel, with a mean 142,940 annual visits to the ED over the last 5 years^3^. Of these visits, about 7,200 a year are referred to a psychiatrist, i.e., about 600 visits monthly. During the COVID-19 outbreak, we noticed a change in the psychiatric ED visits, both in number and in nature. We set out to investigate these trends. Our aim was to assess the effect of the pandemic on patients' health seeking behavior and mental health needs. We were interested in investigating whether the numbers and characteristics of psychiatric referrals to the ED were different during the early COVID-19 period, compared to the same months in previous years with the possible influence of the social isolation, financial worries, unemployment and uncertainty. We hypothesized that the number of visits will decrease due to fears of infection, and that there will be an increase in depression, anxiety, suicidality, and self-harm. We postulate that if there will a decrease in referrals, and neglection of psychiatric emergencies, this in turn will result in higher suicidality rates in the future. The paper will try to approach suicidality in the early pandemic period, by comparing to rates of suicide and suicide attempts in previous years.

## Methods

We examined the number of psychiatric visits to the emergency department in Rambam Health Care Campus, during the months March and April 2020, the period of the first COVID-19 mandatory quarantine in Israel. Those months were compared with identical months in the years 2018 and 2019, such that overall, 6 months were analyzed. An examination of the Jewish calendar revealed that major holidays were included in the months examined during the 3-year period. This could have an effect on number and nature of visits- several studies have demonstrated that there is a change in ED visits patterns along the holidays, a slight decrease before the holiday and an increase thereafter (9). Having the holidays all in those months neutralized the effect of religious holidays on ED visits.

In addition to the total number of referrals, ICD-9 diagnoses were retrieved. Since the Israeli health care system currently uses the ICD-9 codes, and since chapter 5 (mental disorder) of the ICD-9 comprises many categories, we decided to divide the diagnoses into 11 categories with respect to ICD-9:

1. Consultation for observation.
2. Psychosis (as in new onset psychosis and not as a manifestation of a previously diagnosed disease).
3. Schizophrenia and schizophrenic spectrum disorders.
4. Post-traumatic stress disorder.
5. Anxiety and related disorders.
6. Mood disorders.
7. Adjustment disorders.
8. Personality disorders.
9. Suicide, self-harm, and related disorders.
10. Substance abuse and related disorders.
11. Non-psychiatric diagnoses (diagnoses that are not included in chapter 5 of the ICD-9).

It is noteworthy that “Consultation for observation” is a diagnosis given for visits in which the examined patient is without a major psychiatric disorder, brought for psychiatric evaluation either for safety evaluation, or for those whose diagnosis is unclear (in our hospital, mostly soldiers and detainees).

Medical records were retrieved using the MD Clone program by our hospital's computer team. Inclusion criteria were age over 18 and referral to psychiatric consultation in the ED.

A prior suicide attempt is a robust risk factor for future complete suicide (10). Due to the importance of suicide attempts on future outcomes, we built a larger sample cohort, which included patients from a smaller more rural hospital with community-based clinics. Our aim here was to broaden the perspective in regard to the COVID-19 influence on suicide attempts. We speculated that persons might avoid attending a larger hospital in fear that it would pose a greater risk of COVID-19. We expected that such persons might thus be more likely to seek help in a smaller hospital or community clinic. Thus, in our investigation of suicide attempts, suicidal ideation, self-harm and related disorders, we considered data from Emek Medical Center (a non-referral hospital serving 700,000 residents), and Meuhedet health maintenance organization (HMO), the third largest HMO in Israel, serving over one million residents throughout the country, and about 250,000 in our area.

The study was approved by the local bioethical committee of Rambam Health Care Campus, Haifa, Israel, as well as the additional centers.

Statistical significance was set a priori at < 0.05 (two-tailed), with all analyses conducted using IBM SPSS Statistics version 24 (IBM, Armonk, NY). The chi square test was used to compare categorical data between groups, with results presented as count (percentage).

## Results

**Figure 1:**
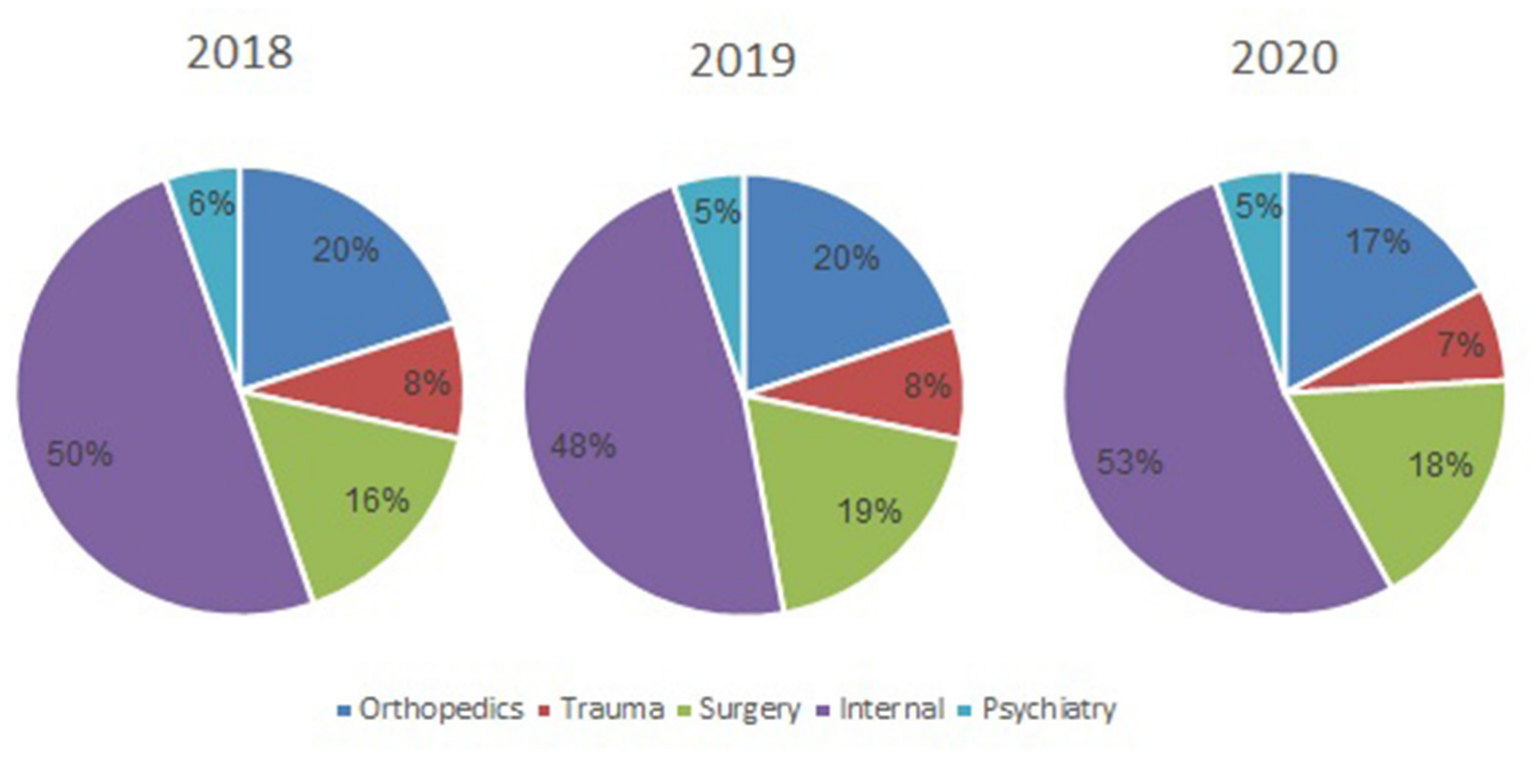
The proportion of psychiatric consultations of the total number of visits to the emergency department, by year.

During March-April 2020, the total number of visits to the ED was 9,136. Of these, 462 (5.1%) were referred to psychiatric consultation. This compares with referrals to psychiatric consultation of 656/12,309 (5.3%) and 652/12,733 (5.1%) during 2018 and 2019, respectively. The number of ED visits that resulted in referrals to psychiatric consultation did not differ significantly between 2018 and 2019. However, a 30% decrease in the referrals to psychiatric consultation in the ED was observed in 2020 (Figure 1).

Table 1 presents the numbers and proportions of patients with each of the 11 diagnostic classifications delineated above, during March-April 2018–2019 and 2020. Compared to 2018–2019, in the COVID-19 period, March-April 2020, the proportions of the following diagnostic classifications were higher: anxiety disorders (14.5 vs. 5.4%, *p* < 0.001), personality disorders (6.7 vs. 3.2%, *p* = 0.001), psychosis (9.5 vs. 6.7%, *p* = 0.049), and post-traumatic stress disorder (PTSD) (3.2 vs. 1.5%, *p* = 0.023). Compared to 2018–2019, in the COVID-19 period, March-April 2020, proportions were lower for adjustment disorder (5.8 vs. 8.9%, *p* = 0.036) and consultation for observation (11.7 vs. 31.6%, *p* < 0.001). Differences were not observed between 2018–2019 and 2020 in the proportions of diagnoses for substance abuse and related disorders, mood disorders, non-psychiatric diagnoses, schizophrenia, suicide, and self-harm disorders.

**Table 1:** The numbers and proportions of patients with each of 11 diagnostic classifications during March-April 2018–2019 and during March-April 2020 (the COVID-19 lockdown).

**Diagnosis classification**	**Frequency**		***p*****-value**
	**2018+2019**	**2020**	
	***n*** **= 1,308**	***n*** **= 462**	
Adjustment disorder	117 (8.9%)	27 (5.8%)	0.036
Anxiety and related disorders	71 (5.4%)	67 (14.5%)	< 0.001
Substance abuse and related disorders	76 (5.8%)	35 (7.6%)	0.178
Mood disorders	109 (8.3%)	43 (9.3%)	0.521
Consultation for observation	413 (31.6%)	54 (11.7%)	< 0.001
Non-psychiatric diagnosis	236 (18%)	90 (19.5%)	0.493
Personality disorders	42 (3.2%)	31 (6.7%)	0.001
Psychosis	88 (6.7%)	44 (9.5%)	0.049
Post-traumatic stress disorder	20 (1.5%)	15 (3.2%)	0.023
Schizophrenia	102 (7.8%)	44 (9.5%)	0.246
Suicide and self-harm related disorders	34 (2.6%)	12 (2.6%)	0.998

**Figure 2:**
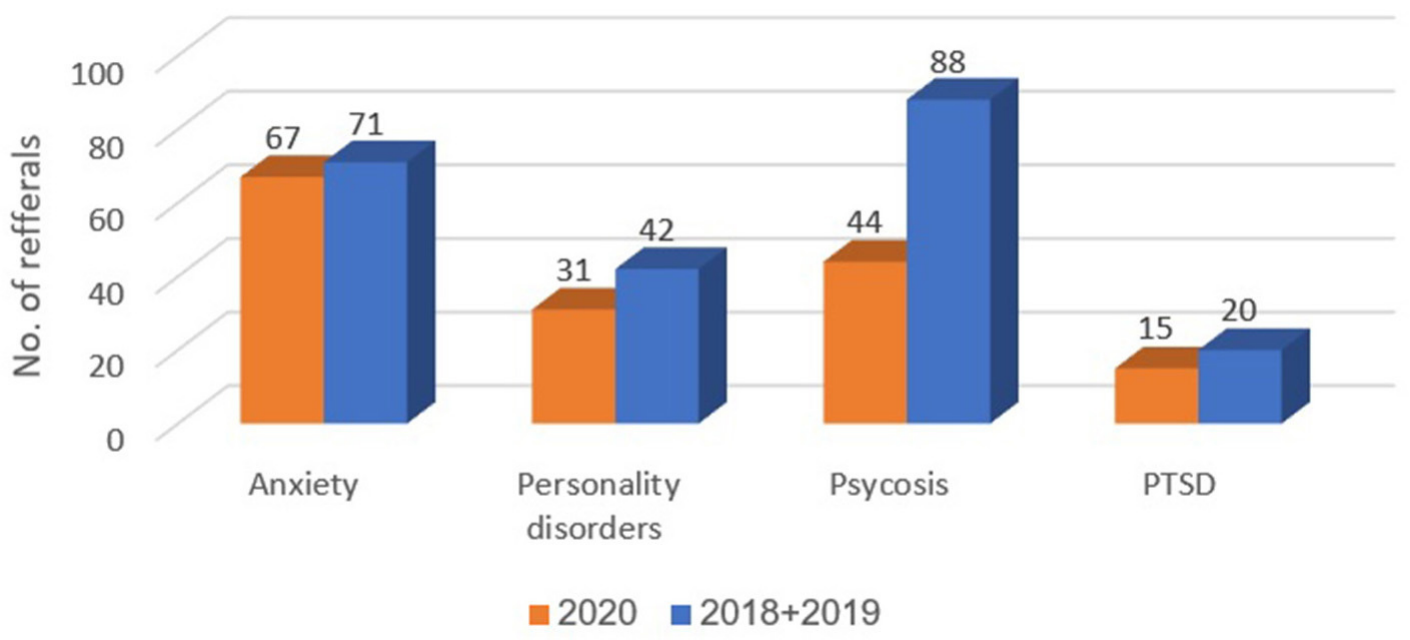
Representation of diagnoses that increased in proportion of occurrence.

**Figure 3:**
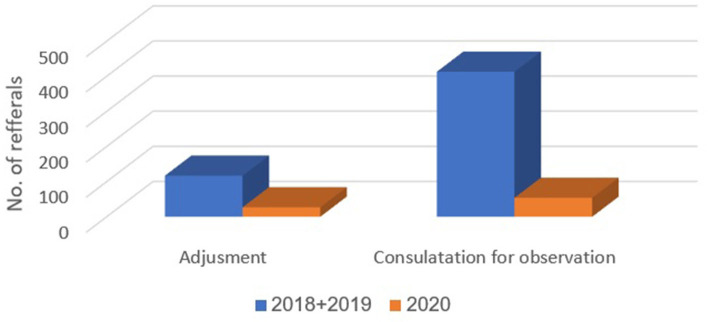
Representation of diagnoses that decreased in proportion of occurrence.

**Figure 4:**
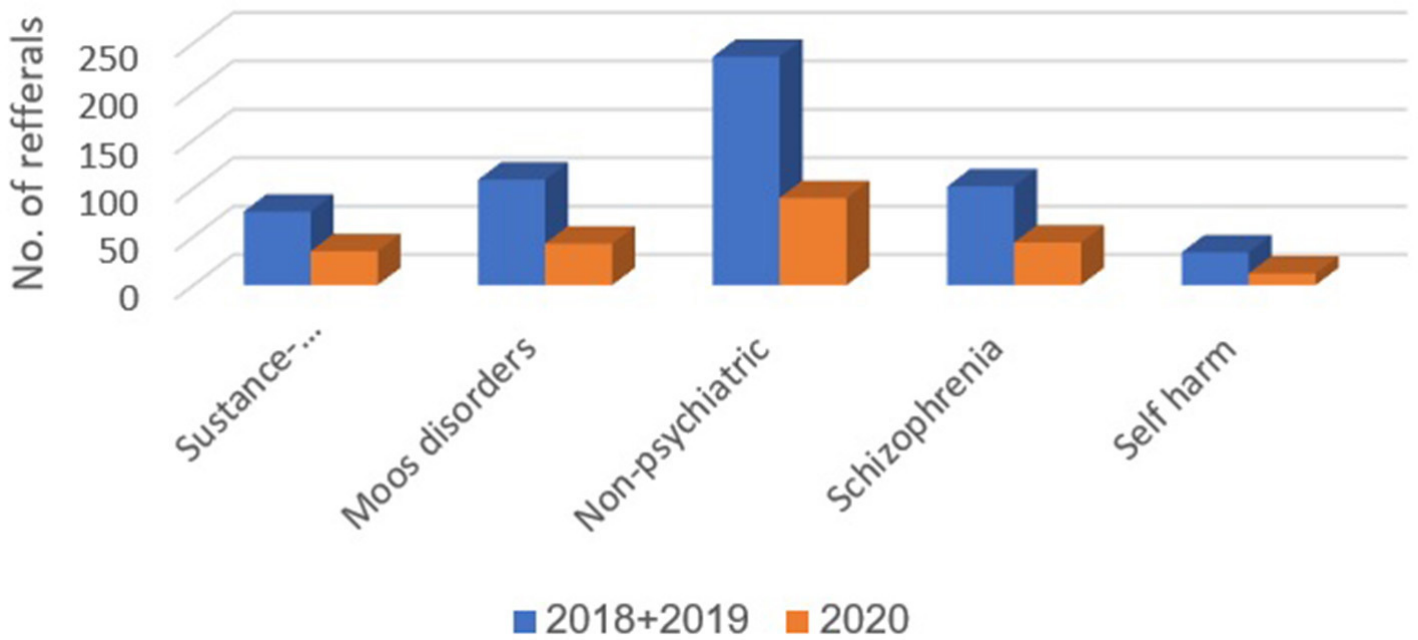
Representation of diagnoses that remained the same in proportion of occurrence.

In Emek Medical Center, 67 referrals to psychiatric consultation regarding suicide and self-harm were recorded during March-April 2018 and 2019 (30 and 37 referrals, respectively). In contrast, 23 referrals were recorded during March-April 2020 (a 30% decrease). In Meuhedet Health Services, 12 referrals due to suicide and self-harm were recorded during March-April 2018 and 2019; and only four during 2020 (a 34% decrease). Figures 2–4 show trends in Psychiatric visits during COVID-19 outbreak comparing with reviewed months in 2019, 2018.

## Discussion

Our results show a dramatic decrease during the COVID-19 period in the number of psychiatric visits to the ED. This paralleled the decrease in overall visits to the ED, such that the proportion of psychiatric referrals of the total number of ED visits was similar in the 3 years, about 5%. The lower number of visits could be explained by the fear of arriving at a general hospital that also treats patients with COVID-19. Moreover, during the mandatory quarantine, public transportation was shut down after 8 PM, and strict rules in the hospital concerning escorts (only one companion per visitor was allowed). While the roads to the hospital ED were obviously open, the quarantine as a state of mind might have influenced some persons to stay at home, even during psychiatric crises. The similar proportions of psychiatric and non-psychiatric ED visits suggest that the fear of coming to the ED and the possible exposure to COVID- 19 or breaking the quarantine affected persons with psychiatric issues as it did persons with other health issues. We presume that the visits to the ED represented urgent needs that could not be postponed, and at least some neglect in medical needs—psychiatric and physical, regarding less urgent needs.

An increase in the diagnosis of anxiety disorder is intuitively understood in the period of uncertainty and loneliness governed by the quarantine, and fears of health and financial crises (5.4% during 2018 and 2019, compared with 14.5% in 2020). Under such circumstances, it is only natural that people would feel more anxious, and would seek medical help and reassurance. Due to the face that outpatients' clinics were closed at the time, these patients sought medical help in the ER.

The increase in the diagnosis of personality disorder is also understood (3.2% during 2018 and 2019 compared with 6.7% during 2020). The diagnosis of personality disorder represents a long-standing hardship, usually combined with rigid coping skills. Thus, during the quarantine, the changes in society, including the closure of ambulatory services, and loss of jobs and stability, were disruptive. For some, such as persons with schizoid personality disorder, staying distanced from surrounding society could be perceived as helpful. However, for those in need of ongoing support and close attention of mental health professionals, such as persons with borderline personality disorder, being alone, or in the proximity of stressing and ambivalent family members who may be in distress themselves, is not an option. Thus, seeking help in the ED is inevitable.

An increase in the diagnosis of psychosis (6.7% during 2018 and 2019 compared with 9.5% during 2020) could be attributed to two major factors: one is the unveiling of an underlying psychotic disorder that could not be ignored due to the long time spent in close quarters with family members. The second is the global, local and personal stress caused by the COVID-19, and the accompanying social and economic uncertainty.

External and internal stress can increase the chance of a diagnosis of PTSD or impair the state of a person treated for PTSD. These patients can be difficult to treat, even under ideal conditions, let alone in times of turmoil, anxiety, and uncertainty. As with persons with personality disorders, the closing of treatment facilities and the use of telemedicine is inadequate for these patients, and they were compelled to seek emergency assistance. Furthermore, isolation in living quarters can exacerbate outbursts of a person with emotion regulation difficulties, thus prompting family members to bring individuals with such difficulties to the ED.

The pandemic was assumed to lead to an increase in the proportion of referrals of patients with adjustment disorders, anxiety or depressed features- out of the total number of patients referred to psychiatric consultation in the ED. However, the fear of contracting the infection in the ED apparently decreased substantially the numbers of ED visits of people with psychiatric problems that were considered manageable, such as adjustment disorder, or those in need of observation. The fewer than expected ED visits could also be attributed to the massive organizing of civilian groups of mutual responsibility and support groups, who made house visits to elderly people, and to those in need due to loss of work or other hardships caused by the pandemic. We assume that this effect may diminish as the pandemic continues. The reopening of treatment centers at the end of the lockdown precludes untangling the specific effects of the various factors on psychiatric ED visits. Another possible explanation to the fact that adjustment disorder referrals have gone down in percentage, is that patients that were previously diagnosed as suffering from an adjustment disorder are now diagnosed as anxiety disorder given the length and severity of illness due to the pandemic.

We expected a higher rate of depression among persons arriving to the ED during the pandemic, especially in light of the closure of community clinics. The steady rather than increasing rate of depression could be attributed to the same civilian group organizations mentioned above, and the fear of infection in the ED. Presumably, lack of mental health treatment over time could lead to detrimental consequences in the future, including an increased suicide rate. Another diagnosis that showed no significant change was the number of referrals regarding substance use disorder. We assume that in the time of data collection, the long-term damage of substance use were yet to be seen. We postulate that this will change over time and there will be in increase of referrals of such type in the near future.

One of the most interesting findings of this research was the lack of change in the proportions of referrals that concerned suicide attempts and self-harm. The decreased visits to the more rural hospital and the community clinics managed by the health maintenance organization support the lack of increase in help-seeking behavior related to suicidality and self-harm. This seemingly unexpected finding could be explained by the hypothesis of the “pulling together effect” (11). Accordingly, a community-shared crisis with its bonding elements could be a protective factor against self-harm. We believe that such effect will dissipate as the economic crises intensifies and the neglected treatment of depression continues.

Our study includes several limitations. As retrospective research, data were collected from medical files, signed by several doctors, hence there was no unity. Nonetheless, we expect that the retrieval of data over 3 years would mitigate this bias. Our study is looking at the proportion of different illnesses between different years rather than absolute numbers, those percentages should be regarded with caution (e.g., there me be an actual increase of a diagnosis of a decrease in others, making it take a larger slice of the pie).

COVID-19 has emerged abruptly, and has altered almost every aspect of our lives. In the field of emergency psychiatry, we found a change in health seeking behaviors in our population, marked by an overall decrease in ED psychiatric visits. Among these visits, the nature of complaints also changed. As this is only the first- shock reaction, we can assume that the trends will change, and move toward a much greater need for psychiatric interventions, higher depression and suicidality rates. We believe that the mental health systems should further investigate the meaning of the changing demands so the trends will be recognized and the system ready.

## Data Availability Statement

The raw data supporting the conclusions of this article will be made available by the authors, without undue reservation.

## Ethics Statement

The studies involving human participants were reviewed and approved by bioethical committee of Rambam Health Care Campus, Haifa, Israel. Written informed consent for participation was not required for this study in accordance with the national legislation and the institutional requirements.

## Author Contributions

YP and SH: conceptualization, data curation, investigation, methodology, and writing—original draft. IM and AD: conceptualization, data curation, methodology, project administration, and writing—review and editing. LG, AL, and SB: data curation, formal analysis, and writing—review and editing. IS: data curation, formal analysis, and software. YN: project administration and data curation. BB: data curation, formal analysis, writing—review and editing, and validation. RE: data curation, investigation, formal analysis, software, and writing—review and editing. EF: conceptualization, data curation, investigation, methodology, project administration, supervision, visualization, and writing—original draft. All authors contributed to the article and approved the submitted version.

## Conflict of Interest

The authors declare that the research was conducted in the absence of any commercial or financial relationships that could be construed as a potential conflict of interest.

## Footnotes

^1^Available online at: https://www.who.int/emergencies/diseases/novel-coronavirus-2019/situation-reports/

^2^Available online at: https://govextra.gov.il/ministry-of-health/corona/corona-virus

^3^Available online at: https://www.rambam.org.il/emergency_room/
